# 6-(4-Nitro­phen­oxy)hexa­nol

**DOI:** 10.1107/S160053680901722X

**Published:** 2009-05-14

**Authors:** Muhammad Saif Ullah Khan, Zareen Akhter, Michael Bolte, Sajjad A. Cheema, Humaira M. Siddiqi

**Affiliations:** a Department of Chemistry, Quaid-i-Azam University, Islamabad 45320, Pakistan; b Institut für Anorganische Chemie, J. W. Goethe-Universität Frankfurt, Max-von-Laue-Str. 7, 60438 Frankfurt/Main, Germany; c Alchemist SAC, PO Box 321, Faisalabad 38000, Pakistan

## Abstract

The title compound, C_12_H_17_NO_4_, features an almost planar mol­ecule (r.m.s. deviation for all non-H atoms = 0.070 Å). All methyl­ene C—C bonds adopt an anti­periplanar conformation. In the crystal structure the mol­ecules lie in planes parallel to (1
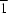
2) and the packing is stabilized by O—H⋯O hydrogen bonds.

## Related literature

For background material on polymers and their properties, see: Manners (1999 ▶); Jarzabek *et al.* (1999 ▶) Schab-Balcerzak *et al.* (2002 ▶); Choi *et al.* (2004 ▶); Hsiao & Lin (2004 ▶); Shao *et al.* (2007 ▶); Shockravi *et al.* (2007 ▶); Yin *et al.* (1998 ▶). For studies on a related compound, see: Saeed *et al.* (2008 ▶).
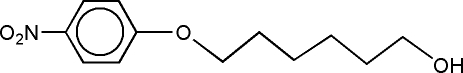


## Experimental

### 

#### Crystal data

C_12_H_17_NO_4_
*M* *_r_* = 239.27
Triclinic, 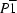

*a* = 5.4410 (7) Å
*b* = 10.2270 (11) Å
*c* = 11.3333 (14) Å
α = 96.993 (9)°
β = 103.818 (10)°
γ = 99.516 (10)°
*V* = 595.34 (12) Å^3^
*Z* = 2
Mo *K*α radiation
μ = 0.10 mm^−1^
*T* = 173 K
0.25 × 0.24 × 0.12 mm

#### Data collection

STOE IPDS II diffractometer
Absorption correction: none
5002 measured reflections
2105 independent reflections
1694 reflections with *I* > 2σ(*I*)
*R*_int_ = 0.074

#### Refinement

*R*[*F* ^2^ > 2σ(*F* ^2^)] = 0.066
*wR*(*F* ^2^) = 0.185
*S* = 1.04
2105 reflections
159 parameters
H atoms treated by a mixture of independent and constrained refinement
Δρ_max_ = 0.31 e Å^−3^
Δρ_min_ = −0.33 e Å^−3^

### 

Data collection: *X-AREA* (Stoe & Cie, 2001 ▶); cell refinement: *X-AREA*; data reduction: *X-AREA*; program(s) used to solve structure: *SHELXS97* (Sheldrick, 2008 ▶); program(s) used to refine structure: *SHELXL97* (Sheldrick, 2008 ▶); molecular graphics: *PLATON* (Spek, 2009 ▶); software used to prepare material for publication: *SHELXL97*.

## Supplementary Material

Crystal structure: contains datablocks I. DOI: 10.1107/S160053680901722X/tk2444sup1.cif

Structure factors: contains datablocks I. DOI: 10.1107/S160053680901722X/tk2444Isup2.hkl

Additional supplementary materials:  crystallographic information; 3D view; checkCIF report

## Figures and Tables

**Table 1:** Hydrogen-bond geometry (Å, °)

*D*—H⋯*A*	*D*—H	H⋯*A*	*D*⋯*A*	*D*—H⋯*A*
O1—H1⋯O4^i^	0.83 (5)	2.10 (5)	2.905 (2)	163 (4)

Symmetry code: (i) 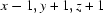.

**Fig. 1.**
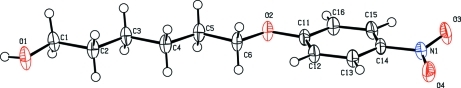
Perspective view of (I) with the atom numbering scheme. Displacement ellipsoids are at the 50% probability level and H atoms are drawn as small spheres of arbitrary radii.

## supplementary crystallographic information

### Comment

Polymers are ubiquitous because of their tremendous processing advantage over ceramics and metals (Manners, 1999). Therefore, much research in recent years has focused upon producing speciality polymers with a better balance of properties (Shockravi *et al.*, 2007). The goal can be achieved by inducing desired modifications in the polymer core structure (Saeed *et al.*, 2008). Flexible linkages such as for the aryl-ether moiety (Shao *et al.*, 2007) and/or methylene spacers (Yin *et al.*, 1998) can be introduced into the macro chain in order to obtain desirable polymers. It has been recognized that the incorporation of an aryl-ether moiety generally imparts enhanced solubility and processability while maintaining the toughness of the polymers (Hsiao & Lin, 2004). Moreover, the addition of aliphatic methylene spacers between the aromatic moieties increases the degree of freedom by reducing the segmental barrier and effectively disrupts potential intermolecular interactions (Schab-Balcerzak *et al.*, 2002). Furthermore, the inclusion of these flexible linkages in the polymer core structure also imparts mesogenic (Choi *et al.*, 2004) and optical properties (Jarzabek *et al.*, 1999) to the resulting polymer. Thus, the final polymer produced by the introduction of these linkages exhibits not an enhancement in its processability but also an improvement in its performance (Jarzabek *et al.*, 1999). The title compound, (I), Fig. 1, is a flexible nitro-alcohol precursor with built-in aliphatic (methylene) groups along with aryl-ether moiety, which was prepared as part of our quest to design and synthesize structurally modified monomers for processable high performance polymers (Saeed *et al.*, 2008).

### Experimental

The title compound (I) was synthesized by Williamson's etherification of 1,6-hexane diol and *p*-nitrochlorobenzene. A three-necked round bottom flask equipped with reflux condenser, thermometer and nitrogen inlet was charged with a suspension of 1,6-hexane diol (2.5 g; 21 mmol) and anhydrous potassium carbonate (2.93 g; 21 mmol) in dimethylformamide (60 ml) and stirred for 30 mins. Then *p*-nitrochlorobenzene (3.33 g; 21 mmol) was added dropwise to the suspension and the resulting mixture was heated to 383 K for 6 h. The reaction mixture was poured into 500 ml of chilled water, cooled to room temperature and the crude product was filtered as a light-yellow solid mass. The product was then washed thoroughly with water, dissolved in ethanol and set aside for crystallization. Yield 74%, m.p. 357 K.

### Refinement

H atoms were geometrically positioned and refined using a riding model with fixed individual displacement parameters [U(H) = 1.2 *U*_eq_(C)] using a riding model with C—H(aromatic) = 0.95Å and *C*—*H*(methylene) = 0.99 Å. The hydroxyl-H was refined freely; O—H = 0.83 (5) Å.

### Crystal data

**Table:**

C_12_H_17_NO_4_	*Z* = 2
*M**_r_* = 239.27	*F*(000) = 256
Triclinic, *P*1	*D*_x_ = 1.335 Mg m^−^^3^
Hall symbol: -P 1	Mo *K*α radiation, λ = 0.71073 Å
*a* = 5.4410 (7) Å	Cell parameters from 4859 reflections
*b* = 10.2270 (11) Å	θ = 3.8–25.6°
*c* = 11.3333 (14) Å	µ = 0.10 mm^−^^1^
α = 96.993 (9)°	*T* = 173 K
β = 103.818 (10)°	Plate, yellow
γ = 99.516 (10)°	0.25 × 0.24 × 0.12 mm
*V* = 595.34 (12) Å^3^	

### Data collection

**Table:**

STOE IPDS II two-circle-diffractometer	1694 reflections with *I* > 2σ(*I*)
Radiation source: fine-focus sealed tube	*R*_int_ = 0.074
graphite	θ_max_ = 25.0°, θ_min_ = 3.8°
ω scans	*h* = −6→6
5002 measured reflections	*k* = −12→12
2105 independent reflections	*l* = −13→13

### Refinement

**Table:**

Refinement on *F*^2^	Secondary atom site location: difference Fourier map
Least-squares matrix: full	Hydrogen site location: inferred from neighbouring sites
*R*[*F*^2^ > 2σ(*F*^2^)] = 0.066	H atoms treated by a mixture of independent and constrained refinement
*wR*(*F*^2^) = 0.185	*w* = 1/[σ^2^(*F*_o_^2^) + (0.1186*P*)^2^ + 0.0716*P*] where *P* = (*F*_o_^2^ + 2*F*_c_^2^)/3
*S* = 1.04	(Δ/σ)_max_ = 0.001
2105 reflections	Δρ_max_ = 0.31 e Å^−^^3^
159 parameters	Δρ_min_ = −0.33 e Å^−^^3^
0 restraints	Extinction correction: *SHELXL*, Fc^*^=kFc[1+0.001xFc^2^λ^3^/sin(2θ)]^-1/4^
Primary atom site location: structure-invariant direct methods	Extinction coefficient: 0.029 (8)

### Special details

**Table:**

Geometry. All e.s.d.'s (except the e.s.d. in the dihedral angle between two l.s. planes) are estimated using the full covariance matrix. The cell e.s.d.'s are taken into account individually in the estimation of e.s.d.'s in distances, angles and torsion angles; correlations between e.s.d.'s in cell parameters are only used when they are defined by crystal symmetry. An approximate (isotropic) treatment of cell e.s.d.'s is used for estimating e.s.d.'s involving l.s. planes.
Refinement. Refinement of *F*^2^ against ALL reflections. The weighted *R*-factor *wR* and goodness of fit *S* are based on *F*^2^, conventional *R*-factors *R* are based on *F*, with *F* set to zero for negative *F*^2^. The threshold expression of *F*^2^ > σ(*F*^2^) is used only for calculating *R*-factors(gt) *etc*. and is not relevant to the choice of reflections for refinement. *R*-factors based on *F*^2^ are statistically about twice as large as those based on *F*, and *R*- factors based on ALL data will be even larger.

### Fractional atomic coordinates and isotropic or equivalent isotropic displacement parameters (Å^2^)

**Table:**

	*x*	*y*	*z*	*U*_iso_*/*U*_eq_	
N1	0.7262 (3)	0.23792 (17)	−0.02359 (16)	0.0296 (4)	
O1	0.1593 (3)	1.17994 (15)	0.78559 (16)	0.0418 (5)	
H1	0.058 (9)	1.200 (5)	0.826 (4)	0.107 (15)*	
O2	0.2906 (3)	0.47709 (14)	0.32079 (13)	0.0310 (4)	
O3	0.6941 (3)	0.11523 (15)	−0.04278 (16)	0.0428 (5)	
O4	0.8558 (3)	0.30971 (15)	−0.07523 (14)	0.0385 (5)	
C1	0.0903 (4)	1.0372 (2)	0.7533 (2)	0.0321 (5)	
H1A	−0.0980	1.0087	0.7153	0.039*	
H1B	0.1349	0.9954	0.8278	0.039*	
C2	0.2376 (4)	0.9931 (2)	0.6628 (2)	0.0313 (5)	
H2A	0.4248	1.0151	0.7046	0.038*	
H2B	0.2081	1.0444	0.5938	0.038*	
C3	0.1586 (4)	0.8432 (2)	0.6108 (2)	0.0297 (5)	
H3A	0.1895	0.7916	0.6795	0.036*	
H3B	−0.0287	0.8209	0.5693	0.036*	
C4	0.3067 (4)	0.8006 (2)	0.51947 (19)	0.0304 (5)	
H4A	0.2806	0.8547	0.4525	0.036*	
H4B	0.4935	0.8207	0.5620	0.036*	
C5	0.2263 (4)	0.6526 (2)	0.46311 (19)	0.0307 (5)	
H5A	0.2555	0.5975	0.5292	0.037*	
H5B	0.0395	0.6314	0.4205	0.037*	
C6	0.3777 (4)	0.6177 (2)	0.37264 (19)	0.0310 (5)	
H6A	0.3505	0.6729	0.3064	0.037*	
H6B	0.5645	0.6365	0.4150	0.037*	
C11	0.4034 (4)	0.4260 (2)	0.23531 (18)	0.0259 (5)	
C12	0.5917 (4)	0.5012 (2)	0.19465 (19)	0.0292 (5)	
H12	0.6492	0.5948	0.2255	0.035*	
C13	0.6963 (4)	0.4383 (2)	0.10791 (19)	0.0289 (5)	
H13	0.8253	0.4886	0.0785	0.035*	
C14	0.6111 (4)	0.3028 (2)	0.06527 (18)	0.0260 (5)	
C15	0.4211 (4)	0.2257 (2)	0.10414 (19)	0.0305 (5)	
H15	0.3649	0.1321	0.0734	0.037*	
C16	0.3156 (4)	0.2890 (2)	0.18899 (19)	0.0295 (5)	
H16	0.1824	0.2389	0.2160	0.035*	

### Atomic displacement parameters (Å^2^)

**Table:**

	*U*^11^	*U*^22^	*U*^33^	*U*^12^	*U*^13^	*U*^23^
N1	0.0340 (9)	0.0249 (9)	0.0317 (9)	0.0102 (7)	0.0134 (8)	−0.0030 (7)
O1	0.0510 (10)	0.0244 (8)	0.0567 (11)	0.0068 (7)	0.0347 (9)	−0.0067 (7)
O2	0.0334 (8)	0.0262 (8)	0.0349 (8)	0.0050 (6)	0.0182 (7)	−0.0067 (6)
O3	0.0568 (11)	0.0237 (9)	0.0532 (10)	0.0128 (7)	0.0276 (8)	−0.0055 (7)
O4	0.0477 (10)	0.0330 (9)	0.0428 (9)	0.0097 (7)	0.0290 (8)	0.0012 (6)
C1	0.0358 (11)	0.0231 (11)	0.0404 (12)	0.0063 (8)	0.0196 (9)	−0.0037 (8)
C2	0.0319 (11)	0.0290 (12)	0.0348 (11)	0.0060 (9)	0.0171 (9)	−0.0043 (9)
C3	0.0291 (10)	0.0280 (11)	0.0339 (11)	0.0088 (8)	0.0142 (9)	−0.0041 (8)
C4	0.0298 (10)	0.0289 (11)	0.0340 (11)	0.0071 (9)	0.0153 (9)	−0.0037 (8)
C5	0.0314 (11)	0.0314 (12)	0.0315 (11)	0.0092 (9)	0.0149 (9)	−0.0029 (8)
C6	0.0366 (11)	0.0260 (11)	0.0322 (11)	0.0080 (9)	0.0168 (9)	−0.0057 (8)
C11	0.0271 (10)	0.0270 (11)	0.0256 (10)	0.0098 (8)	0.0109 (8)	−0.0022 (8)
C12	0.0338 (11)	0.0227 (10)	0.0317 (11)	0.0054 (8)	0.0141 (8)	−0.0040 (8)
C13	0.0327 (10)	0.0247 (10)	0.0316 (11)	0.0061 (8)	0.0155 (9)	−0.0013 (8)
C14	0.0292 (10)	0.0246 (11)	0.0258 (10)	0.0098 (8)	0.0108 (8)	−0.0030 (8)
C15	0.0361 (11)	0.0208 (10)	0.0346 (11)	0.0066 (8)	0.0128 (9)	−0.0039 (8)
C16	0.0316 (10)	0.0242 (11)	0.0343 (11)	0.0038 (8)	0.0160 (9)	−0.0011 (8)

### Geometric parameters (Å, °)

**Table:**

N1—O3	1.223 (2)	C4—H4A	0.9900
N1—O4	1.228 (2)	C4—H4B	0.9900
N1—C14	1.457 (2)	C5—C6	1.506 (3)
O1—C1	1.425 (2)	C5—H5A	0.9900
O1—H1	0.83 (5)	C5—H5B	0.9900
O2—C11	1.362 (2)	C6—H6A	0.9900
O2—C6	1.441 (2)	C6—H6B	0.9900
C1—C2	1.516 (3)	C11—C12	1.381 (3)
C1—H1A	0.9900	C11—C16	1.395 (3)
C1—H1B	0.9900	C12—C13	1.392 (3)
C2—C3	1.525 (3)	C12—H12	0.9500
C2—H2A	0.9900	C13—C14	1.373 (3)
C2—H2B	0.9900	C13—H13	0.9500
C3—C4	1.522 (3)	C14—C15	1.385 (3)
C3—H3A	0.9900	C15—C16	1.381 (3)
C3—H3B	0.9900	C15—H15	0.9500
C4—C5	1.517 (3)	C16—H16	0.9500
			
O3—N1—O4	122.51 (16)	C6—C5—H5A	109.5
O3—N1—C14	119.32 (17)	C4—C5—H5A	109.5
O4—N1—C14	118.16 (16)	C6—C5—H5B	109.5
C1—O1—H1	105 (3)	C4—C5—H5B	109.5
C11—O2—C6	117.46 (15)	H5A—C5—H5B	108.0
O1—C1—C2	108.19 (17)	O2—C6—C5	108.46 (17)
O1—C1—H1A	110.1	O2—C6—H6A	110.0
C2—C1—H1A	110.1	C5—C6—H6A	110.0
O1—C1—H1B	110.1	O2—C6—H6B	110.0
C2—C1—H1B	110.1	C5—C6—H6B	110.0
H1A—C1—H1B	108.4	H6A—C6—H6B	108.4
C1—C2—C3	113.07 (17)	O2—C11—C12	123.92 (18)
C1—C2—H2A	109.0	O2—C11—C16	115.44 (17)
C3—C2—H2A	109.0	C12—C11—C16	120.64 (17)
C1—C2—H2B	109.0	C11—C12—C13	119.14 (19)
C3—C2—H2B	109.0	C11—C12—H12	120.4
H2A—C2—H2B	107.8	C13—C12—H12	120.4
C4—C3—C2	112.49 (18)	C14—C13—C12	119.37 (19)
C4—C3—H3A	109.1	C14—C13—H13	120.3
C2—C3—H3A	109.1	C12—C13—H13	120.3
C4—C3—H3B	109.1	C13—C14—C15	122.40 (18)
C2—C3—H3B	109.1	C13—C14—N1	118.70 (18)
H3A—C3—H3B	107.8	C15—C14—N1	118.90 (18)
C5—C4—C3	113.70 (17)	C16—C15—C14	118.02 (18)
C5—C4—H4A	108.8	C16—C15—H15	121.0
C3—C4—H4A	108.8	C14—C15—H15	121.0
C5—C4—H4B	108.8	C15—C16—C11	120.41 (18)
C3—C4—H4B	108.8	C15—C16—H16	119.8
H4A—C4—H4B	107.7	C11—C16—H16	119.8
C6—C5—C4	110.92 (17)		
			
O1—C1—C2—C3	−173.95 (17)	C12—C13—C14—C15	0.9 (3)
C1—C2—C3—C4	179.61 (18)	C12—C13—C14—N1	−178.77 (17)
C2—C3—C4—C5	−178.18 (17)	O3—N1—C14—C13	164.72 (18)
C3—C4—C5—C6	179.28 (17)	O4—N1—C14—C13	−14.2 (3)
C11—O2—C6—C5	179.40 (16)	O3—N1—C14—C15	−14.9 (3)
C4—C5—C6—O2	−179.08 (16)	O4—N1—C14—C15	166.11 (19)
C6—O2—C11—C12	−1.1 (3)	C13—C14—C15—C16	−0.1 (3)
C6—O2—C11—C16	179.11 (16)	N1—C14—C15—C16	179.60 (18)
O2—C11—C12—C13	179.23 (18)	C14—C15—C16—C11	−1.3 (3)
C16—C11—C12—C13	−1.0 (3)	O2—C11—C16—C15	−178.36 (18)
C11—C12—C13—C14	−0.4 (3)	C12—C11—C16—C15	1.8 (3)

### Hydrogen-bond geometry (Å, °)

**Table:**

*D*—H···*A*	*D*—H	H···*A*	*D*···*A*	*D*—H···*A*
O1—H1···O4^i^	0.83 (5)	2.10 (5)	2.905 (2)	163 (4)

Symmetry codes: (i) *x*−1, *y*+1, *z*+1.
